# Usefulness of bevacizumab-induced hypertension in patients with metastatic colorectal cancer: an updated meta-analysis

**DOI:** 10.18632/aging.101478

**Published:** 2018-06-21

**Authors:** Chun-Jing Zhang, Shu-Ying Zhang, Chun-Di Zhang, Chun-Rong Lin, Xue-Yan Li, Qiu-Yan Li, Hai-Tao Yu

**Affiliations:** 1 Department of Biochemistry, Institute of Medical Technology, Qiqihar Medical University, Qiqihar, Heilongjiang, China; 2 Division of Hematology, The Second Affiliated Hospital of Qiqihar Medical University, Qiqihar, Heilongjiang, China; 3 Department of Foreign Languages, Qiqihar Medical University, Qiqihar, Heilongjiang, China; 4 Institute of Polygenic Diseases, School of Medicine and Pharmacy, Qiqihar Medical University, Qiqihar, Heilongjiang, China; 5 Department of Cell Biology and Medical Genetics, Basic Medical College, Qiqihar Medical University, Qiqihar, Heilongjiang, China

**Keywords:** colorectal cancer, bevacizumab, hypertension, usefulness, meta-analysis

## Abstract

We tested the hypothesis that bevacizumab-induced hypertension may be a useful predictor for objective response rate, progression-free and overall survival in patients with metastatic colorectal cancer via a comprehensive meta-analysis. Search process, article selection and data extraction were independently performed by two investigators. Statistical analyses were conducted using the STATA/SE software. Fourteen independent studies and 2292 study subjects were synthesized. Overall relative risk of objective response rate for bevacizumab-induced hypertension was 2.03 (95% confidence interval [CI]: 1.18-3.48, *p*=0.01), with significant heterogeneity and publication bias, whereas unbiased estimate was nonsignificant after considering potentially missing studies. Overall hazard ratio for progression-free survival was 0.58 (95% CI: 0.43-0.77, *p*<0.001), with significant heterogeneity and publication bias, and unbiased estimate was significant (hazard ratio: 0.52, 95% CI: 0.41-0.66, *p*<0.001). Overall hazard ratio for overall survival was 0.51 (95% CI: 0.39-0.65, *p*<0.001), and this estimate was not likely confounded by heterogeneity or publication bias. Subgroup and meta-regression analyses suggested that hypertension grade of controls, sample size, age and gender were possible causes of heterogeneity. Taken together, our findings indicate that bevacizumab-induced hypertension can predict progress-free survival and overall survival in patients with metastatic colorectal cancer, whereas its prediction for objective response rate was nonsignificant.

## Introduction

Colorectal cancer is the third most common cancer in the world, and its global burden is projected to be increased by 60% to over 2.2 million new cases and 11 million deaths by 2030 [1]. Clinical studies indicate that colorectal cancer predominantly metastasizes to the liver and lung [2-4]. It is estimated that approximately one in four patients have metastatic colorectal cancer at initial diagnosis, and almost half of colorectal cancer patients will develop metastases [5]. Over the past decade, considerable advances have been made in the treatment of metastatic colorectal cancer using chemotherapy and effective biotherapy, with great success [6-8]. It is well exemplified by the fact that median survival of metastatic colorectal cancer increased from 5 months to 2 years between 1993 and 2009 [9].

Bevacizumab, a humanized anti-vascular endothelial growth factor (VEGF) monoclonal antibody, is increasingly recognized as standard of care for the treatment of metastatic colorectal cancer in a first-line setting, with reasonable biological implications [10,11]. However, a problem facing global oncologists is that the clinical benefits of bevacizumab are seen in some, but not all, patients with metastatic colorectal cancer [12,13]. Given the enormous economic burden for bevacizumab-based treatment, the identification of a surrogate marker to gauge the usefulness of bevacizumab treatment in metastatic colorectal cancer is of particular importance to select patients who are more likely to benefit from the treatment. Many candidate predictive markers have been tested, and arterial hypertension is one of the most intensively researched [14,15]. Two previous meta-analyses have interrogated the usefulness of hypertension, by showing that bevacizumab-induced hypertension may be a prognostic factor for metastatic colorectal cancer [16,17]. However, an inherent drawback gripping the two meta-analyses is the under-explored heterogeneity and publication bias, likely due to the limited number of studies synthesized. With accumulating data on this subject in recent years, we sought to update the results of two previous meta-analyses by incorporating more studies and providing additional information. Specifically, we examined the hypothesis that bevacizumab-induced hypertension may be a useful predictor for objective response rate and survival outcomes (progression-free and overall survival) in patients with metastatic colorectal cancer.

## RESULTS

### Qualified studies

**Figure 1:**
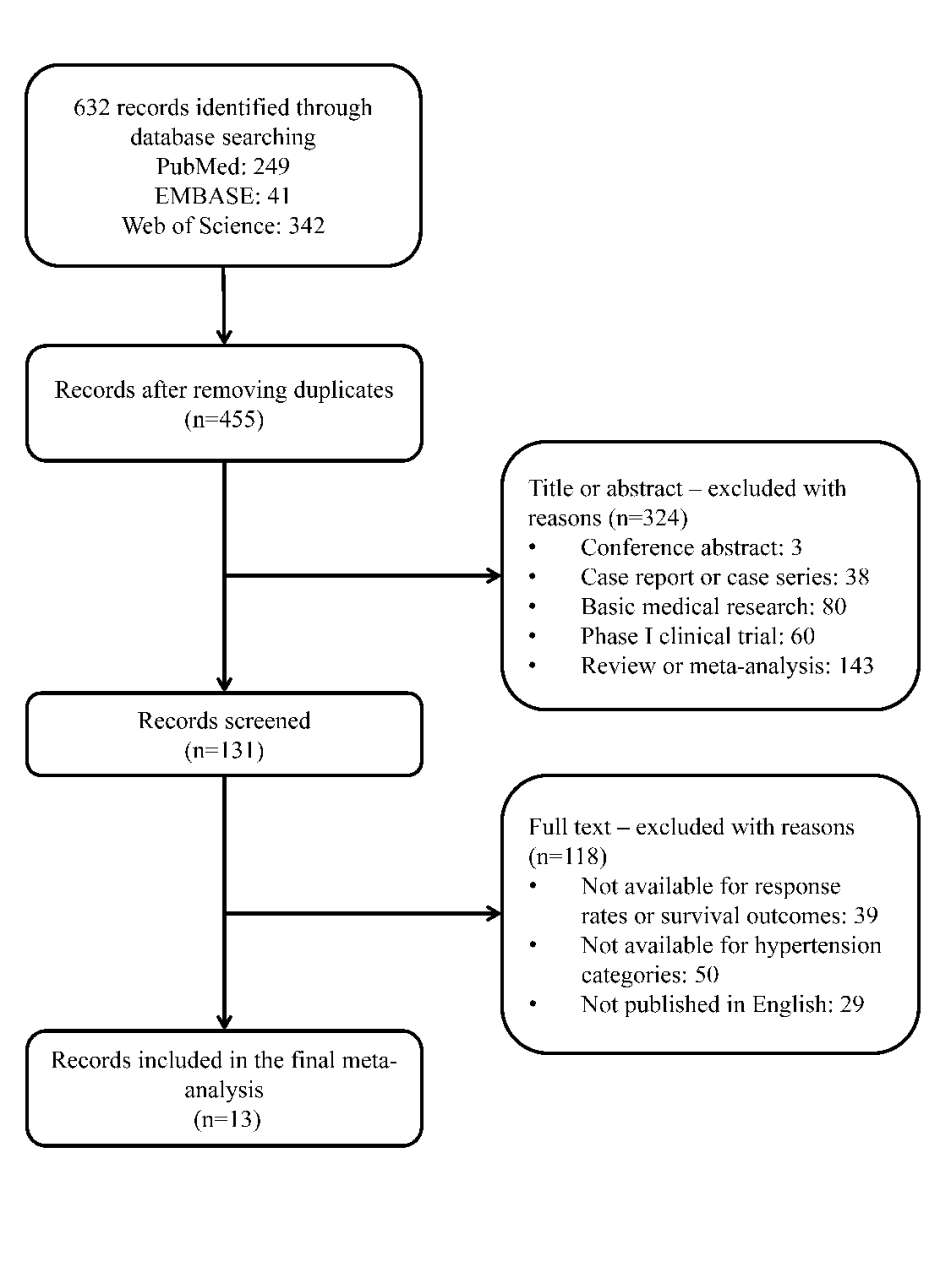
The flowchart for article selection in this meta-analysis.

In total, 632 articles were identified after searching the medical literature, as well as the reference lists of retrieved major articles and reviewers. Of them, only 13 articles including 14 independent studies and 2292 study subjects were qualified for the final analysis [12-15,18-26]. The process for excluding articles with specific reasons was presented as a PRISMA flowchart (Figure 1). In terms of research outcomes, 8 of 14 qualitied studies provided data on objective response rate, 9 studies on progression-free survival and 7 studies on overall survival, when gauging the usefulness of bevacizumab-induced hypertension in patients with metastatic colorectal cancer.

### Baseline characteristics

The baseline characteristics of 14 qualified studies published from 2009 to 2016 are listed in Table 1. Total sample size of each study ranged from 39 to 699. All but one study (in Japan) [25] were conducted in European countries and the United States of America. Ten studies used bevacizumab in the first-line setting [12,14,18-20,22-25]. Bevacizumab dose was either 2.5 mg/kg every week or 5 mg/kg every 2 weeks or 7.5 mg/kg every three weeks. Hypertension was diagnosed using the CTC AE (common terminology criteria; AE: adverse events) version 2.0 or 3.0 or 4.0. Male gender of each study ranged from 50% to 67.1%.

**Table 1:** Characteristics of the 14 selected studies in this meta-analysis.

**Author (year)**	**No. of patients**	**No. of patients with HTN (%)**	**Gender (M/F)**	**Line of treatment**	**Bevacizumab dose**	**Chemotherapy regiments**	**HTN criteria**	**Cut-off point**	**Median PFS (months) HTN/non-HTN**	**Median OS (months) HTN/non-HTN**	**ORR (%) HTN/non-HTN**
Ryanne (2009)	84	36 (42.9)	42/42	First	NA	NA	CTC AE V3.0	Grade = 0	NA	NA	NA
Scartozzi (2009)	39	8 (20.5)	25/14	First	5 mg/kg/2w	FOLFIRI	CTC AE V2.0	Grade < 2	14.5/3.1	NA/15.1	75/32
De Stefano (2011)	74	13 (17.6)	42/32	First	5 mg/kg/2w or 7.5 mg/kg/3w	FOLFIRI, FOLFOX, XELOX, XELIRI, FOLFOXIRI	CTC AE V3.0	Grade = 0	15.1/8.3	35.5/26.7	84.6/42.6
Mir (2011)	119	65 (54.6)	63/56	NA	2.5 mg/kg/w	5-FU-based	CTC AE V3.0	Grade = 0	NA	NA	76.9/79.6
Osterlund (2011)	101	57 (56.4)	54/47	Combined	5 mg/kg/2w or 7.5 mg/kg/3w	FOLFIRI, irinotecan-, oxaliplatin- or 5-FU-based	CTC AE V3.0	Grade = 0	10.5/5.3	25.8/11.7	52.6/45.5
Dewdney (2012)	45	7 (15.6)	NA	First	7.5 mg/kg/3w	CAPOX	CTC AE V3.0	Grade = 0	NA	NA	71/78
Budai (2013)	232	NA	126/106	First	5 mg/kg/2w	modified FOLFIRI	CTC AE V3.0	Grade ≤ 1	NA	NA	NA
Hurwitz (2013)	402	NA	237/165	First	5 mg/kg/2w	IFL	CTC AE V2.0	SBP/DBP increase 20/10 mmHg	NA	NA	NA
Hurwitz (2013)	699	NA	418/281	First	5 mg/kg/2w	FOLFOX-4	CTC AE V3.0	SBP/DBP increase 20/10 mmHg	NA	NA	NA
Morita (2013)	60	16 (26.7)	38/22	First	5 mg/kg/2w	mFOLFOX6, FOLFIRI, sLV5FU2, XELOX	CTC AE V4.0	Grade ≤ 2	NA	NA	NA
Tahover (2013)	181	81 (44.8)	95/86	First	2.5 mg/kg/w	oxaliplatin, 5FU combination, irinotecan, 5FU combination, both combinations	CTC AE V4.0	Grade ≤ 1	17.2/29.9	36.8/NA	NA
Khoja (2014)	50	7 (14)	NA	Combined	NA	tyrosine kinase inhibitor (TKI)	CTC AE V3.0	Grade ≤ 1	10.9/9.4	25.2/21.6	NA
Feliu J (2015)	127	20 (15.7)	78/49	NA	7.5 mg/kg/3w	capecitabine in BECA, oxaliplatin, capecitabine in BECOX	CTC AE V2.0	Grade = 0	NA	NA/16.9	NA
de Sousa (2016)	79	41 (51.9)	53/26	First	5 mg/kg/2w	FOLFIRI or FOLFOX regimen	CTC AE V4.0	Grade ≤ 1	NA	33/21	NA

Abbreviations: HTN: hypertension; M: male; F: female; NA: not available; SBP: systolic blood pressure; DBP: diastolic blood pressure; ORR: objective response rate; OS: overall survival; PFS: progression free survival; CTC: common terminology criteria; AE: adverse events.

### Objective response rate

**Figure 2:**
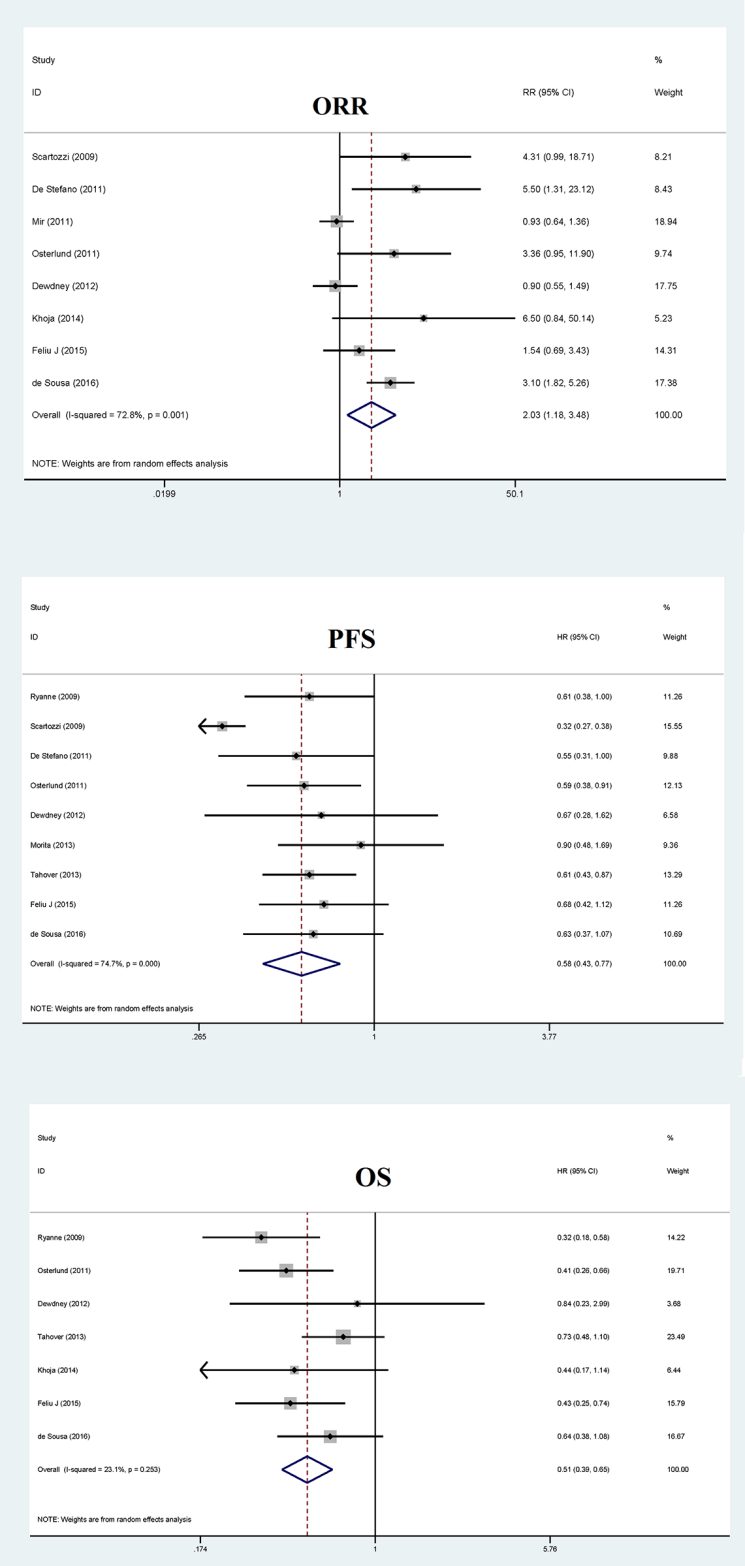
Overall forest plots of objective response rate (ORR), progression-free survival (PFS) and overall survival (OS) for bevacizumab-induced hypertension in patients with metastatic colorectal cancer.

As shown in Figure 2, pooled relative risk of objective response rate for bevacizumab-induced hypertension was 2.03 (95% confidence interval [CI]: 1.18 – 3.48, *p*=0.01), whereas this risk was clouded by the significance of between-study heterogeneity (*I*^2^: 77.1%).

Heterogeneity sources were explored using both subgroup analysis and meta-regression analysis. In subgroup analysis, bevacizumab dose, sample size and hypertension grade of controls may account for the presence of significant heterogeneity based on significant differences in stratified relative risk (Table 2). For example, when analysis was restricted to studies with hypertension grade 1/2 of controls, the risk of objective response rate for bevacizumab-induced hypertension was statistically significant (relative risk: 3.35, 95% CI: 2.06 – 5.44, *p*<0.001) and was not impacted by heterogeneity (*I*^2^: 0.0%), with the effect estimate over twice as much as that in studies with hypertension grade 0 in controls (relative risk: 1.47, 95% CI: 0.82 – 2.64, *p*=0.195, *I*^2^: 69.3%). Moreover, no significance was detected after dividing studies using the median cutoff value of total sample size at 77, in spite of divergent difference in risk estimates. In univariate meta-regression analysis, gender was identified as a significant source of heterogeneity for overall response rate (*p*=0.037).

**Table 2:** Subgroup analyses of response rates and survival outcomes for the presence of hypertension in bevacizumab-treated patients with metastatic colorectal cancer.

**Outcomes**	**Groups and subgroups**	**Studies**	**Sample size**	**RE**	**95% CI**	***P* value**	***I*^2^ (%)**
ORR	Bevacizumab dose			RR			
	2.5 mg/kg/w	1	119	0.93	0.64 – 1.36	0.716	NA
	5 mg/kg/2w	2	118	3.22	1.95 – 5.30	<0.001	0.0
	5 mg/kg/2w or 7.5 mg/kg/3w	2	175	4.17	1.61 – 10.77	0.003	0.0
	7.5 mg/kg/3w	2	172	1.10	0.62 – 1.94	0.753	37.3
	Hypertension diagnosis						
	CTC AE V2.0	2	166	2.13	0.83 – 5.46	0.115	31.8
	CTC AE V3.0	5	389	1.82	0.85 – 3.93	0.126	76.5
	CTC AE V4.0	1	79	3.10	1.82 – 5.26	<0.001	NA
	Hypertension cut-off point in controls						
	Grade 0	5	466	1.47	0.82 – 2.64	0.195	69.3
	Grade 1/2	3	168	3.35	2.06 – 5.44	<0.001	0.0
	No. of patients						
	< 77	4	208	3.07	0.69 – 13.76	0.142	82.5
	≥ 77	4	426	1.84	0.87 – 3.89	0.110	81.1
PFS	Bevacizumab dose			HR			
	2.5 mg/kg/w	1	181	0.61	0.43 – 0.87	0.006	NA
	5 mg/kg/2w	3	178	0.54	0.27 – 1.06	0.075	86.3
	5 mg/kg/2w or 7.5 mg/kg/3w	2	175	0.58	0.41 – 0.82	0.002	0.0
	7.5 mg/kg/3w	2	172	0.68	0.44 – 1.05	0.078	0.0
	Hypertension diagnosis						
	CTC AE V2.0	2	166	0.45	0.21– 0.95	0.037	88.1
	CTC AE V3.0	4	304	0.60	0.46 – 0.78	<0.001	0.0
	CTC AE V4.0	3	320	0.66	0.51 – 0.86	0.002	0.0
	Hypertension cut-off point in controls						
	Grade 0	5	431	0.62	0.49 – 0.78	<0.001	0.0
	Grade 1/2	4	359	0.55	0.33 – 0.77	0.021	86.2
	No. of patients						
	< 79	4	218	0.54	0.30 – 0.95	0.032	79.0
	≥ 79	5	572	0.62	0.51 – 0.76	<0.001	0.0
OS	Bevacizumab dose			HR			
	2.5 mg/kg/w	1	181	0.73	0.48 – 1.10	0.128	NA
	5 mg/kg/2w	1	79	0.64	0.38 – 1.08	0.096	NA
	5 mg/kg/2w or 7.5 mg/kg/3w	1	101	0.41	0.26 – 0.66	<0.001	NA
	7.5 mg/kg/3w	2	172	0.47	0.29 – 0.79	0.004	0.0
	Hypertension diagnosis						
	CTC AE V2.0	1	127	0.43	0.25 – 0.74	0.002	NA
	CTC AE V3.0	4	280	0.40	0.29 – 0.56	<0.001	0.0
	CTC AE V4.0	2	260	0.69	0.50 – 0.96	0.026	0.0
	Hypertension cut-off point in controls						
	Grade 0	4	357	0.41	0.30 – 0.55	<0.001	0.0
	Grade 1/2	3	310	0.66	0.49 – 0.90	0.008	0.0
	No. of patients						
	< 84	3	174	0.61	0.39 – 0.94	0.025	0.0
	≥ 84	4	493	0.47	0.32 – 0.67	<0.001	52.4

Abbreviations: ORR: objective response rate; PFS: progression free survival; OS: overall survival; RE: risk estimate; 95% CI: 95% confidence interval; OR: odds ratio; HR: hazard ratio; *I*^2^: inconsistency index; CTC: common terminology criteria; AE: adverse events; NA: not available.

**Figure 3:**
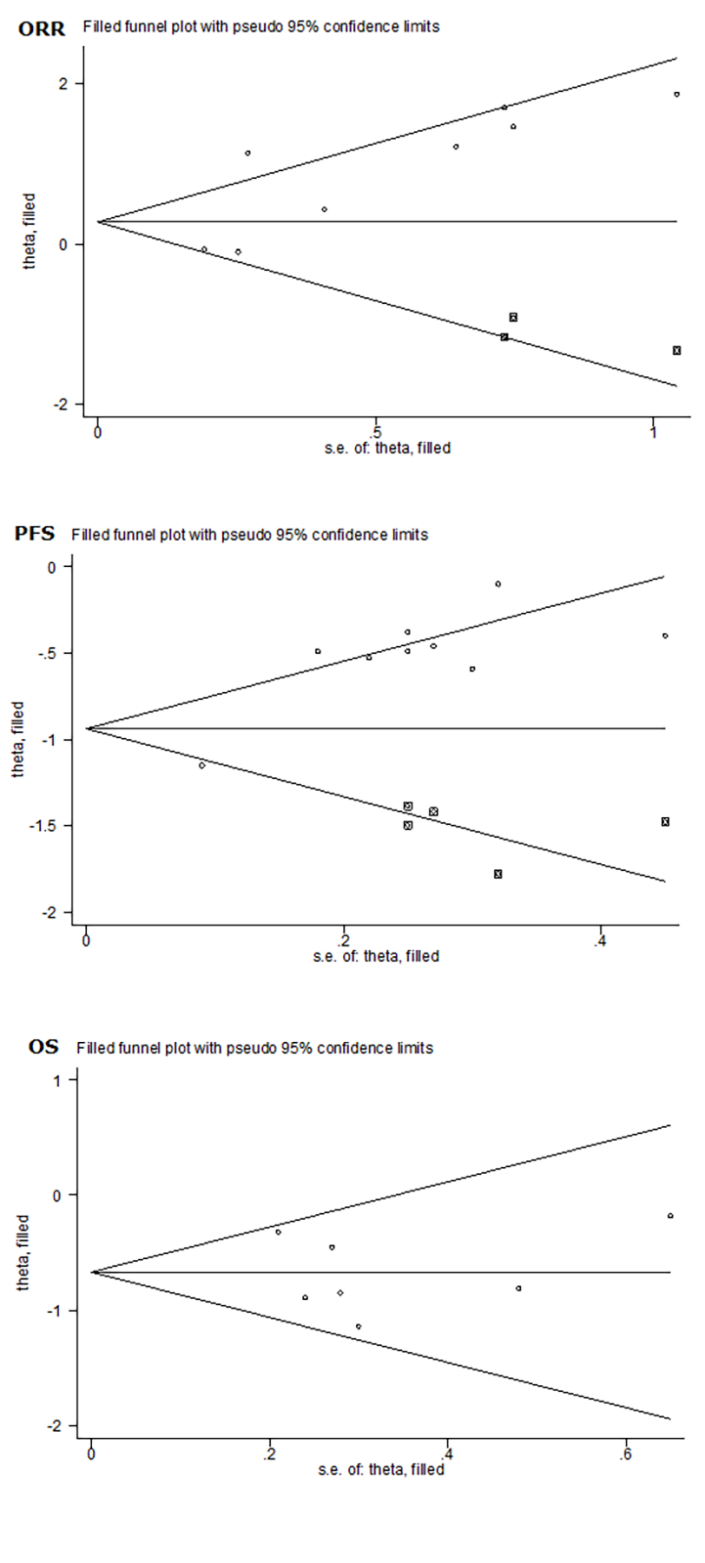
Overall funnel plots of objective response rate (ORR), progression-free survival (PFS) and overall survival (OS) for bevacizumab-induced hypertension in patients with metastatic colorectal cancer.

Publication bias was evaluated using both filled funnel plots (Figure 3) and Egger’s tests. Three missing studies were needed to ensure the symmetry of filled funnel plot, signaling a high probability of publication bias as reflected by Egger’s test (*p*=0.047). Analysis of incorporating the three missing studies showed that the unbiased relative risk of response rate was 1.55, which did not deviate significantly from 1 (95% CI: 0.95 – 2.54, *p*=0.081). Further cumulative meta-analysis indicated a stable trend in the risk estimates of objective response rate for bevacizumab-induced hypertension, as presented in Supplementary Figure S1.

### Progression-free survival

Analysis of all qualified studies showed that pooled hazard ratio of bevacizumab-induced hypertension for progression-free survival was 0.58 (95% CI: 0.43 – 0.77, *p*<0.001), with significant heterogeneity (*I*^2^: 74.7%) (Figure 2).

Subgroup analysis showed that hypertension grade of controls and sample size may confound the prediction of bevacizumab-induced hypertension in patients with metastatic colorectal cancer based on the differences in hazard ratio between subgroups (Table 2). The risk estimates were more obvious in subgroups with hypertension grade 0 in controls and with total sample size over the median cutoff value 79 of total sample size, and were not impacted by heterogeneity (both *I*^2^: 0.0%). In meta-regression analysis, age and gender were identified as significant confounders for the prediction of bevacizumab-induced hypertension for progression-free survival (*p*=0.011 and 0.002, respectively).

As shown in Figure 3, filled funnel plot for progression-free survival detected five potentially missing studies, and the associated Egger’s test was remarkably significant (*p*=0.001). After adjusting for the five missing studies, the hazard ratio of progression-free survival was still significant (hazard ratio: 0.52, 95% CI: 0.41 – 0.66, *p*<0.001). In cumulative meta-analysis, a stable trend in risk estimates was noted for progression-free survival (SSupplementary Figure S1).

### Overall survival

The pooled hazard ratio for overall survival of bevacizumab-induced hypertension was 0.51 (95% CI: 0.39 – 0.65, *p*<0.001), and this estimate was not likely confounded by heterogeneity (*I*^2^: 23.1%), as presented in Figure 2. Subgroup analysis showed that sample size and hypertension grade of controls were possible causes of heterogeneity in view of the differences in hazard ratio between subgroups (Table 2). The risk estimates were reinforced when analysis was done using studies with hypertension grade 0 in controls (hazard ratio: 0.41, *p*<0.001) and with total sample size over the median cutoff value of total sample size at 84 (hazard ratio: 0.47, *p*<0.001). Meta-regression analysis showed that age and gender might be other causes of heterogeneity (*p*=0.024 and 0.015, respectively).

No missing study was reported by filled funnel plot in Figure 3, and no evidence of publication bias was detected by the Egger’s test (*p*=0.83). Risk estimates were stabilized in cumulative meta-analysis, as shown in SSupplementary Figure S1.

### Trial sequential analysis

**Figure 4:**
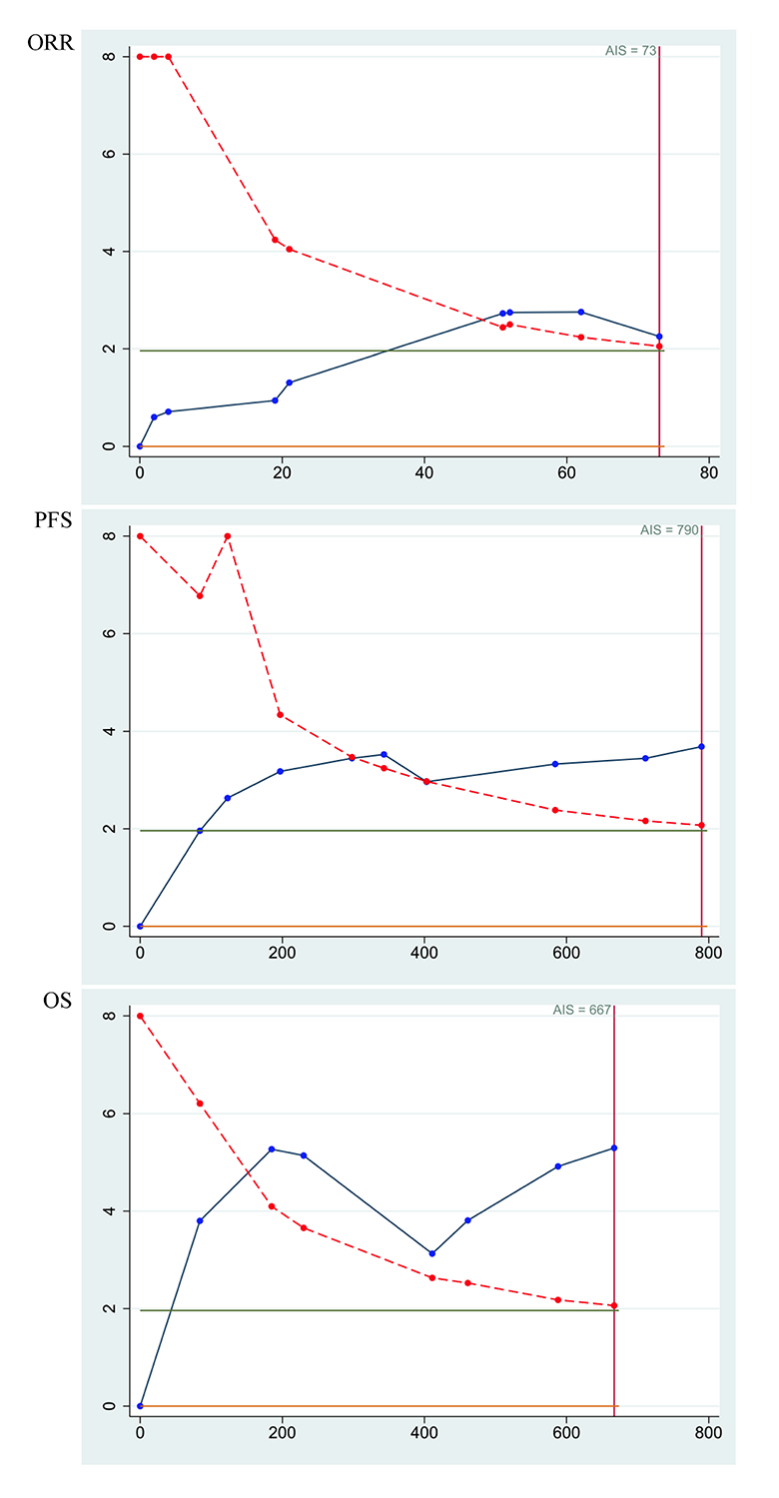
Trial sequential analysis of objective response rate (ORR), progression-free survival (PFS) and overall survival (OS) for bevacizumab-induced hypertension in patients with metastatic colorectal cancer.

Finally, trial sequential analysis was employed to minimize random errors for objective response rate, progression-free survival and overall survival, respectively (Figure 4). The three cumulative z-curves were noticed to cross trial sequential monitoring boundaries prior to reaching the required information sizes, which suggested adequate cumulative evidence and the robustness of our conclusions.

## DISCUSSION

The aim of this present study was to update the results of two previous meta-analyses [16,17] by incorporating more studies and analyzing data more comprehensively. The key finding of this study was that bevacizumab-induced hypertension can significantly predict progress-free survival and overall survival in patients with metastatic colorectal cancer, whereas its prediction for objective response rate was nonsignificant. Moreover, our findings indicated that sample size and hypertension grade of controls, as well as age and gender, may be possible causes of between-study heterogeneity. To the authors’ knowledge, this study is thus far the largest report for gauging the usefulness of bevacizumab-induced hypertension in patients with metastatic colorectal cancer.

It is widely recognized that VEGF is a key mediator of angiogenesis and an effective biological target for patients with metastatic colorectal cancer, and its overexpression can accelerate tumor progression and metastatic spread of colorectal cancer [27,28]. Also, VEGF signaling cascade can lead to the suppression of nitric oxide production in endothelial cells, which, in turn, results in vasoconstriction and decrease in sodium renal excretion, with an ultimate end point of hypertension [29-32]. Bevacizumab is targeted at VEGF and blocks it from binding to its receptors, therefore impairing angiogenesis and detaining tumor growth and metastasis [33,34]. Hence, it is reasonable to propose that hypertension is a promising indicator for the clinical benefits of bevacizumab in treating patients with metastatic colorectal cancer.

Previously, two meta-analyses examined the validity of using hypertension to predict response rate and survival of patients with metastatic colorectal cancer, and both studies consistently demonstrated that bevacizumab-induced hypertension was associated with significant improvement in objective response rate, progression-free survival and overall survival [16,17]. Differing from the findings of two previous meta-analyses, we only confirmed the predictive role of bevacizumab-related hypertension in survival outcomes, and failed to manifest a significant contribution to objective response rate. The reasons behind this discrepancy may be multifold. One may be related to the inclusion criteria, as only articles published in English language were analyzed in this study, and by contrast the two previous meta-analyses involved two articles published in Japanese language. Another reason lied in the possible existence of publication bias, as our primary results in objective response rate were statistically significant, just as the two meta-analyses did, whereas the probability of publication bias was high and taking the impact of potential missing studies into consideration remarkably weakened the prediction of bevacizumab-induced hypertension for objective response rate. The third reason may be the insufficient power of previous studies, as we have replenished three new articles. The fourth reason may be due to unadjusted residual confounding, as our subgroup and meta-regression analyses indicated that bevacizumab dose, hypertension grade of controls, sample size and gender were possible causes of between-study heterogeneity. Importantly, the nonsignificant relationship between bevacizumab-induced hypertension and objective response rate was independent of the sample size involved, further supporting the claim that bevacizumab-induced hypertension may not be a predictor for objective response rate in patients with metastatic colorectal cancer.

Besides objective response rate, we have assessed the association of bevacizumab-induced hypertension with two survival outcomes. Consistent with the results of two previous studies, our findings consolidated the prognostic contribution of bevacizumab-induced hypertension to both progression-free survival and overall survival. However, extending the results of the two studies, we found that sample size and hypertension grade of controls, as well as age and gender, may be possible causes of between-study heterogeneity. In particular, after grouping studies per the median cutoff point of total sample size, the prediction of bevacizumab-induced hypertension for survival outcomes was more obvious when analysis was restricted to the large studies, indicating the robustness of our findings. In fact, the difference between progression-free survival and overall survival is whether the incorporation of patients who get worse. Just because of this difference, another aspect worth noting was that risk magnitude of overall survival was more obvious in studies with hypertension grade 0 of controls than studies with hypertension grade 1/2, whereas this situation was slightly reversed for that of progression-free survival. In other words, the protective effect of bevacizumab indexed by overall survival was more obvious for a lower grade of hypertension, whereas that by progression-free survival was more obvious for a higher grade of hypertension. Generally, the probability of tumor aggressiveness is higher during early treatment of bevacizumab for metastatic colorectal cancer than the late treatment. A higher grade of bevacizumab-induced hypertension may surrogate a longer treatment period. So the findings of this study further demonstrated the usefulness of using hypertension to signal bevacizumab treatment in metastatic colorectal cancer. Moreover, it is not surprising to notice that the prediction of bevacizumab-induced hypertension for survival outcomes was age- and gender-dependent. As evidenced, aging-related methylation can influence the gene expression of key control genes in colorectal cancer and adenoma [35], and hormone replacement therapy was significantly associated with reduced risk of colorectal cancer incidence and improved colorectal cancer-specific survival in female patients [36]. We agree that further studies are needed to obtain the biological proof and confirm the current findings.

Some limitations deserve special considerations for this meta-analysis. Firstly, only articles published in the English language were identified and the exclusion of gray literature from this meta-analysis can lead to the exaggerated estimates of intervention effectiveness [37]. Secondly, some subgroups involved a limited number of studies, and the probability of heterogeneity cannot be further interrogated. Thirdly, the overall sample size may not be sufficient enough to derive more accurate estimates. Fourthly, only the results of objective response rate, progression-free survival and overall survival were synthesized. Fifthly, data on genomic and epigenomic alterations were not available for us, because it is increasingly recognized that colorectal cancer is a molecularly heterogeneous disease. These alterations can help enhance our understanding of potential personalized therapies for molecularly specific colorectal cancer subtypes [38]. Finally, although we have statistically adjusted for potential missing studies, the jury must refrain from drawing a conclusion until future large, well-designed studies reproduce our findings.

Taken together, through a comprehensive analysis of 14 independent studies and 2292 study subjects, our findings indicate that bevacizumab-induced hypertension can predict progress-free survival and overall survival in patients with metastatic colorectal cancer, whereas its prediction for objective response rate was nonsignificant. Meanwhile, several issues from this meta-analysis remain to be clarified, and yet other areas warrant further investigation. If the usefulness of bevacizumab-induced hypertension were successfully validated in the future, it will be clinically important to administrate bevacizumab agents to patients with metastatic colorectal cancer who are more likely to benefit from the treatment.

## MATERIALS AND METHODS

### Research guideline

The conduct of this meta-analysis was consistent with the guidelines in the Preferred Reporting Items for Systematic Reviews and Meta-analyses (PRISMA) statement (SPRISMA checklist: Supplementary Table S1).

### Search strategy

A systematic literature search was conducted in the following electronic bibliographic databases: PubMed (Medline), EMBASE (Excerpta Medica dataBASE) and Web of Science (Science Citation Index and Social Sciences Citation Index). Search strategy was expressed in the Boolean style, that is, (“colon” or “colorectal” or “rectal” or “rectum”) and (“cancer” or “tumor” or “tumour” or “carcinoma” or “neoplasm”) and (“bevacizumab” or “avastin”) and (“hypertension” or “blood pressure”). The literature search was completed on February 17, 2018. The reference lists of two previous meta-analyses [16,17] and retrieved major articles were also checked for potential missing hits. A final reference list of 632 articles was determined.

### Selection criteria

Articles were retained for analysis pending the simultaneous satisfaction of following criteria: (i) metastatic colorectal cancer patients receiving bevacizumab treatment were grouped according to the presence or severity of hypertension determined by the Common Terminology Criteria for Adverse Events (CTC AE); (ii) information on objective response rate or its associated odds ratio, or hazard ratio for progression-free survival or overall survival, or survival curves was available for extraction or inference; (iii) articles were written in the English language. Meanwhile, conference abstract, case report or case series, review or meta-analysis, basic medical research and phase I clinical trial were not included in this study.

Two investigators (Chun-Jing Zhang and Shu-Ying Zhang) of this present study independently assessed the eligibility of all potential articles according to above criteria. A third investigator (Hai-Tao Yu) solved the disagreements, if exist, from literature screening.

### Data extraction

The following variables were extracted from each eligible article: the first author’s surname, published year, country where study was conducted, CTC AE version, combined chemotherapy, bevacizumab dose, age, gender, metastatic position of metastatic colorectal cancer, objective response rate, progression-free survival, overall survival, odds ratio (95% CI) and hazard ratio (95% CI). Survival rate was used to estimate progression-free survival or overall survival from survival curves in case of no available risk estimates by aid of the Engauge Digitizer software Release 4.0.

Data extraction was independently completed by two investigators (Chun-Jing Zhang and Shu-Ying Zhang) of the present study, and a third investigator (Hai-Tao Yu) checked table entries for accuracy by referring to original context.

### Trial sequential analysis

According to a previous publication [39], trial sequential analysis was performed to calculate the simple accrued information size after assuming a significance level of 5% for type I error and 20% for type II error. In addition, a monitoring boundary was also generated in trial sequential analysis.

### Statistical analysis

Odds ratio and its 95% CI for objective response rate, as well as hazard ratio and its 95% CI for progression-free survival or overall survival were calculated between metastatic colorectal cancer patients with and without bevacizumab-induced hypertension.

From a statistical standpoint, in the absence of between-study heterogeneity, effect estimates based on the fixed-effects model and the random-effects model are exactly the same, whereas in the presence of heterogeneity, effect estimates are more reliable based on the random-effects model, relative to the fixed-effects model. Thus, in this meta-analysis, random-effects model using the DerSimonian & Laird method [40,41] was used for effect estimates.

Between-study heterogeneity was quantified using the inconsistency index (*I*^2^) on the basis of the Cochrane Q-test. Heterogeneity is reported to be low if *I*^2^ ranges from 0% to 25%, moderate from 25% to 75% and high from 75% to 100% [42].

Regardless of the magnitude of heterogeneity, subgroup analysis and meta-regression analysis were conducted to assess the impact of categorical and continuous study variables on the overall heterogeneity.

The presence of publication bias, the bias resulting from the possibly unpublished studies due to the negative results or extreme deviations from previous results, was visually appraised by filled funnel plots. The statistical evidence of publication bias was assessed by the Egger’s test, a weighted regression test that can help justify the asymmetry of funnel plots. In case of evident publication bias, a trim and fill method was employed to derive an unbiased estimate after considering potentially missing studies. In addition, to evaluate whether later studies impacted previous studies, a cumulative meta-analysis was conducted accordingly.

Above statistical analyses were completed using the STATA/SE software Release 11.2.

## Supplementary Material

Supplementary File
